# Clinical characteristics of pediatric noninfectious uveitis and risk factors for severe disease: a single-center study

**DOI:** 10.1007/s10067-024-07072-6

**Published:** 2024-07-27

**Authors:** Lutfiye Koru, Fehim Esen, Ozlem Turkyilmaz, Elif Kucuk, Feray Kaya, Zelal Aydin, Fatih Haslak, Kubra Ozturk

**Affiliations:** 1 Department of Pediatric Rheumatology, Istanbul Medeniyet University, Istanbul, Turkey; 2 Department of Ophthalmology, Istanbul Medeniyet University, Istanbul, Turkey

**Keywords:** Disease severity, Immunosuppressive therapy, Pediatrics, Uveitis

## Abstract

**Objectives:**

We aimed to present the demographic, clinical, laboratory, and treatment data of children with non-infectious uveitis and to evaluate the risk factors for the development of complications and the need for biological treatment.

**Method:**

Patients diagnosed with non-infectious uveitis in childhood and followed up for at least 1 year were included in the study. Demographic data, including age, gender, age at diagnosis, uveitis in first-degree relatives, and rheumatologic diseases, were obtained retrospectively from medical records. The presence of complications or the need for biologic therapy was considered a composite outcome suggesting severe disease.

**Results:**

The study included 123 patients (female: *n* = 59, 48%). The mean age at diagnosis was 14.89 ± 4.86 years. Uveitis was symptomatic in 104 patients (84.6%). Approximately one-quarter of the patients had at least one rheumatic disease (*n* = 35, 28.5%), the most common being juvenile idiopathic arthritis. Thirty-three patients (26.8%) had anti-nuclear antibody positivity. Biologic agents were needed in 60 patients (48.8%). Complications developed in 14 patients (11.4%). Early age at disease onset (aOR, 0.875; 95% C.I. 0.795–0.965, *p* = 0.007) and female gender (aOR, 2.99; 95% C.I. 1.439–6.248, *p* = 0.003) were significantly associated with the need for biologic treatment, while Behçet’s disease (BD) was strongly associated with uveitis-related complications (aOR, 14.133; 95% C.I. 2.765–72.231, *p* = 0.001).

**Conclusion:**

We suggest that among pediatric patients with non-infectious uveitis, females, those with an early age of disease onset, and those with BD need to be closely monitored due to a significantly increased risk of severe disease.

**Table:** 

**Key Points** *• Limited data exist on the clinical course of non-infectious uveitis in children and the associated risk factors for severe disease.* *• Our study reveals that nearly a quarter of pediatric patients with non-infectious uveitis also have a rheumatic disease.* *• Among pediatric patients diagnosed with non-infectious uveitis, we observed an increased risk of severe disease in those with an earlier onset age, in female patients, and in those diagnosed with Behçet’s disease.*

## Introduction

Pediatric uveitis is a serious and rare condition characterized by inflammation of the uveal components of the eye, including the iris, ciliary body, and retina [1]. Idiopathic uveitis accounts for 65–90% of all pediatric uveitis cases [2, 3]. Pediatric uveitis may be associated with various systemic diseases such as juvenile idiopathic arthritis (JIA), Behçet’s disease (BD), tubulointerstitial nephritis uveitis (TINU), sarcoidosis, and vasculitis [4–6]. JIA is the most common systemic disease associated with cases of pediatric uveitis that cannot be attributed to an infectious cause [7].

The main symptoms of uveitis are pain, redness, photophobia, and blurred vision [8]. Because uveitis in children is often insidious and children are less likely to express themselves, the diagnosis of the disease is delayed [8]. As a consequence of delayed diagnosis, uveitis is more likely to lead to complications such as cataracts and band keratopathy in children than in adults [9, 10].

Treatment of uveitis typically includes topical or systemic corticosteroids and conventional disease-modifying antirheumatic drugs (cDMARDs). Biologic therapies have proven to be effective in patients who cannot be controlled with conventional treatments [9].

Limited research has been conducted on non-infectious uveitis in children, and the number of patients included in previous studies was relatively small. The primary aim of our study was to analyze the demographic, laboratory, and systemic disease characteristics of a large pediatric patient population with non-infectious uveitis. Additionally, we aimed to investigate the treatments administered, patients’ responses to drugs, and complications associated with uveitis. Our secondary goal was to identify potential risk factors that may predict the need for biological treatment or the likelihood of experiencing uveitis-related complications.

## Materials and methods

### Patients and data collection

Our study comprised individuals diagnosed with non-infectious uveitis before the age of 21, who experienced the first uveitis attack before the age of 18, and had a minimum of 1 year of follow-up duration. This criterion was based on the guidelines and arrangements in our country that allow us to follow-up these patients until they reach the age of 21.

The demographic, clinic, and laboratory data were obtained from the pediatric rheumatology department’s medical records retrospectively. The ophthalmology department in same center, which specializes in uveitis, provided the data containing eye examination findings. Anatomical classification of uveitis and disease course (acute, chronic, or remission) were classified according to Standardization of Uveitis Nomenclature (SUN) [11]. Underlying systemic diseases, extraocular findings, treatments prescribed for uveitis, complications, and laboratory findings at admission including antinuclear antibody (ANA), human leukocyte antigen-27 (HLA-B27), rheumatoid factor (RF), erythrocyte sedimentation rate (ESR), C-reactive protein (CRP), and platelet count (PLT) were also recorded.

The diagnosis of JIA was made according to the International League of Rheumatology Societies (ILAR) diagnostic criteria for JIA [12]. Pediatric Behçet’s diagnostic criteria were used to make the diagnosis of BD [13]. The diagnoses of familial Mediterranean fever (FMF) and non-hereditary, periodic fever, aphthous, pharyngitis, and adenitis (PFAPA) were made according to the Eurofever/PRINTO clinical classification criteria for PFAPA and hereditary recurrent fevers [14]. The diagnosis of sarcoidosis and tubulointerstitial nephritis and uveitis (TINU) was based on clinical, laboratory, screening, and renal biopsy findings [15, 16].

This study was conducted in compliance with the Helsinki Declaration as well as local laws and regulations. Because of the retrospective nature of the study, obtaining written informed consent from the patients was not required. Ethics committee approval (date/no.: 13/12/23–2023/0919) was obtained from the Istanbul Medeniyet University, Ethics Committee of Clinical Trials.

### Terms and definitions

Non-infectious uveitis was defined as patients not affected by infectious diseases that can cause uveitis, such as toxoplasmosis, toxocariasis, tuberculosis, and viral infections (e.g., herpes simplex virus or cytomegalovirus) [17]. The presence of complications including cataract, synechia, glaucoma, and band keratopathy or the need for biological treatment was considered a composite outcome, and the patients with this composite outcome were considered to have severe disease.

### Statistical analysis

The statistical analysis was executed using SPSS for Windows, version 26.0 (SPSS Inc., Chicago, IL). The continuous variables were exhibited as either the mean ± standard deviation or the median (minimum–maximum), depending on their distribution, which was established using the Kolmogorov–Smirnov test. The assessment of categorical variables was carried out using the chi-square test or Fisher’s exact test when applicable. The associations between continuous variables and categoric variables were assessed using the Mann–Whitney *U* test when abnormally distributed and Student’s *t* tests when normally distributed.

The risk factors of biological requirement and uveitis-related complications identified as the risk factors for severe disease were assigned by using binary logistic regression analysis for both. While model 1 was set for biological requirement, model 2 was for uveitis-related complications. The variables that were found to be significant in comparing tests and confounding factors were included in these models. Gender, presence of rheumatologic disease, ANA positivity, and uveitis type were considered to be confounding factors. Since all BD was panuveitis, uveitis type was not considered a confounding factor in model 2.

Omnibus test and Hosmer–Lemeshow tests were used as model fitting test for binary logictic regression analysis.The significance level employed to determine statistical significance was set at *p* < 0.05, and Prism software (version 8, GraphPad Software, San Diego, California) was utilized to analyze and graph the data.

## Results

### Demographic, laboratory, and underlying systemic diseases data

The average age of 123 patients with non-infectious uveitis was 14.89 ± 4.86 years and 48% (*n* = 59) was female. The median age at onset of uveitis symptoms was 11.72 (1.96–20.01) years. Uveitis was symptomatic in 104 (84.6%) of the patients in our cohort. Bilateral uveitis was more common than unilateral uveitis (82.1% vs 17.9%). According to the anatomical localization of uveitis, 97 patients (78.9%) had anterior uveitis, 11 (8.9%) had intermediate uveitis, 1 (0.8%) had posterior uveitis, and 14 (11.4%) had panuveitis. ANA was positive in 33 (26.8%) of the patients. The median CRP, and the median erythrocyte sedimentation rate (ESR) levels were 2.71 (0.08–94) mg/L and 11 (2–91) mm/hour at admission, respectively. Nearly a quarter of patients (*n* = 35, 28,5%) had at least one type of rheumatic disease, and the most common one was JIA (*n* = 19, 15.4%). Detailed data are given in Table 1.

**Table 1:** Demographic, laboratory, underlying systemic disease, treatment, uveitis-related complication, and disease course data

Characteristic	
Female gender (*n*, %)	59 (48%)
Age (year) (mean. ± SD)	14.89 ± 4.86
Uveitis	
Starting age (median (min–max))	11.72 (1.96–20.01)
Symptomatic (*n*, %)	104 (84.6%)
Unilateral (*n*, %)	22 (17.9%)
Bilateral (*n*, %)	101 (82.1%)
Type	
Anterior (*n*, %)	97 (78.9%)
Intermediate (*n*, %)	11 (8.9%)
Posterior (*n*, %)	1 (0.8%)
Panuveitis (*n*, %)	14 (11.4%)
Extraocular finding (*n*, %)	48 (33.1%)
Arthritis (*n*, %)	21 (17.1%)
Mucocutaneous (*n*, %)	21 (17.1%)
GIS (*n*, %)	2 (1.6%)
CNS (*n*, %)	2 (1.6%)
Renal (*n*, %)	9 (7.3%)
Vascular (*n*, %)	2 (1.6%)
Respiratory (*n*, %)	2 (1.6%)
Rheumatic disease (*n*, %)	35 (28.5%)
FMF (*n*, %)	1 (0.8%)
PFAPA (*n*, %)	1 (0.8%)
JIA (*n*, %)	19 (15.4%)
Behçet (*n*, %)	7 (5.7%)
TINU (*n*, %)	8 (6.5%)
Sarcoidosis (*n*, %)	1 (0.8%)
Family history	
Uveitis in the family (*n*, %)	4 (3.3%)
RD in the family (*n*, %)	11 (8.9%)
Laboratory	
ANA positivity (*n*, %)	33 (26.8%)
ESR (median (min–max))	11 (2–91)
CRP (mg/L) (median (min–max))	2.71 (0.008–94)
Platelets (median (min–max))	287.000 (173.000–670.000)
Treatment	
Systemic steroids (*n*, %)	84 (68.3%)
cDMARD (*n*, %)	97 (78.9%)
Methotrexate (*n*, %)	88 (71.5%)
Sulfasalazine (*n*, %)	6 (4.9%)
AZA (*n*, %)	10 (8.1%)
Cyclosporine (*n*, %)	3 (2.4%)
bDMARD (*n*, %)	60 (48.8%)
Adalimumab (*n*, %)	56 (45.5%)
Infliximab (*n*, %)	7 (5.7%)
Tocilizumab (*n*, %)	1 (0.8%)
Surgery (*n*, %)	6 (4.9%)
Complications (*n*, %)	14 (11.4%)
Band keratopathy (*n*, %)	3 (2.4%)
Synechia (*n*, %)	1 (0.8%)
Glaucoma (*n*, %)	3 (2.4%)
Cataract (*n*, %)	10 (8.1%)
Disease course	
Remission (*n*, %)	22 (17.9%)
Acute (*n*, %)	24 (19.5%)
Chronic (*n*, %)	77 (62.6%)

*ANA* antinuclear antibody, *AZA* azathioprine, *bDMARD* biological disease-modifying antirheumatic drug, *cDMARD* conventional disease-modifying antirheumatic drug, *CNS* central nervous system, *CRP* C-reactive protein, *ESR* erythrocyte sedimentation rate, *FMF* familial Mediterranean fever, *GIS* gastrointestinal system, *JIA* juvenile idiopathic arthritis, *PFAPA* periodic fever, aphthous, pharyngitis and adenitis, *RD* rheumatic disease, *TINU* tubulointerstitial nephritis and uveitis

### Treatment options and uveitis-related complications

All patients in our study received steroid eye drops and 68.3% (*n* = 84) also received systemic steroid treatment. Following the diagnosis of uveitis, 78.9% (*n* = 97) of the patients were treated with cDMARD. The most preferred cDMARD was methotrexate (71.5%). 48.8% of patients (*n* = 60) received biologic treatment. The most preferred biological DMARDs (bDMARDs) were tumor necrosis factor-α (TNF-α) blockers with adalimumab most commonly prescribed (45.5%). In 26 patients (21.1%), neither cDMARD nor bDMARD treatment was required, and uveitis attacks could be controlled with topical steroids only. Almost one tenth of the patients (11.4%, *n* = 14) of the patients experienced uveitis-related complications, and the most common was cataract (*n* = 10). Only three patients required surgical intervention. Detailed data are available in Table 1.

### Comparisons between the patients with and without complications

The uveitis disease onset was significantly earlier in the patients who experienced complications (10.37 (2.92–16.91) years *vs.* 11.80 (1.96–20.01) years; *p* = 0.013). Furthermore, BD was significantly more common in the patients with complications (28.6% vs. 2.8%; *p* < 0.001). While 4 of 7 patients with BD and 2 of 19 patients with JIA developed complications, none of the patients with other rheumatic disease had complications. Detailed data for both groups are presented in Table 2.

**Table 2:** Comparisons between the patients with and without ocular complications

	Patients with ocular complications (*n* = 14)	Patients without ocular complications (*n* = 109)	*p value*
Female gender (*n*, %)	8 (57.1%)	51 (46.8%)	0.656
Age at diagnosis (year) (mean. ± SD)	10.54 ± 4.4.78	11.14 ± 3.84	0.594
Uveitis			
Age at baseline (median (min–max))	10.37 (2.92–16.91)	11.80 (1.96–20.01)	**0.013**
Symptomatic (*n*, %)	11 (78.6%)	93 (85.3%)	0.453
Unilateral (*n*, %)	1 (7.1%)	21 (19.3%)	0.461
Bilateral (*n*, %)	13 (92.9%)	88 (80.7%)	0.461
Type			
Anterior (*n*, %)	7 (50%)	90 (82.6%)	0.064
Intermediate (*n*, %)	3 (21.4%)	8 (7.3%)	0.064
Posterior (*n*, %)	0 (0%)	1 (0.9%)	0.064
Panuveitis (*n*, %)	4 (28.6%)	10 (9.2%)	0.064
Extraocular finding (*n*, %)	7 (50%)	41 (37.6%)	0.546
Arthritis (*n*, %)	3 (21.4%)	18 (16.5%)	0.706
Mucocutaneous (*n*, %)	5 (35.7%)	16 (14.7%)	0.063
GIS (*n*, %)	1 (7.1%)	1 (0.9%)	0.541
CNS (*n*, %)	1 (7.1%)	1 (0.9%)	0.541
Renal (*n*, %)	0 (0%)	9 (8.3%)	0.596
Vascular (*n*, %)	1 (7.1%)	1 (0.9%)	0.541
Respiratory (*n*, %)	1 (7.1%)	1 (0.9%)	0.541
Rheumatic disease (*n*, %)	6 (42.9%)	29 (26.6%)	0.219
FMF (*n*, %)	0 (0%)	1 (0.9%)	1.0
PFAPA (*n*, %)	0 (0%)	1 (0.9%)	1.0
JIA (*n*, %)	2 (14.3%)	17 (15.6%)	1.0
Behçet (*n*, %)	4 (28.6%)	3 (2.8%)	** < 0.001**
TINU (*n*, %)	0 (0%)	8 (7.3%)	0.294
Sarcoidosis (*n*, %)	0 (0%)	1 (0.9%)	1.0
Family history			
Uveitis in the family (*n*, %)	0 (0%)	4 (3.7%)	0.466
RD in the family (*n*, %)	1 (7.1%)	10 (9.2%)	1.0
Laboratory			
ANA positivity (*n*, %)	3 (21.4%)	30 (27.5%)	0.757
ESR (median (min–max))	9.5 (2–66)	12 (2–91)	0.065
CRP (mg/L) (median (min–max))	1.33 (0.42–34)	3 (0–94)	0.514
Platelet (median (min–max))	282.500 (192.000–400.000)	289.000 (173.000–670.000)	0.161
Treatment			
Systemic steroids (*n*, %)	14 (100%)	70 (64.2%)	**0.005**
cDMARD (*n*, %)	13 (92.9%)	84 (77.1%)	0.297
Methotrexate (*n*, %)	10 (71.4%)	78 (71.6%)	1.0
Sulfasalazine (*n*, %)	1 (7.1%)	5 (4.6%)	0.676
AZA (*n*, %)	3 (21.4%)	7 (6.4%)	0.087
Cyclosporine (*n*, %)	1 (7.1%)	2 (1.8%)	0.226
Surgery	6 (42.9%)	0 (0%)	** < 0.001**
bDMARD (*n*, %)	12 (85.7%)	48 (44.0%)	**0.008**
Adalimumab (*n*, %)	10 (71.4%)	46 (42.2%)	0.075
Infliximab (*n*, %)	5 (35.7%)	2 (1.8%)	** < 0.001**
Tocilizumab (*n*, %)	0 (0%)	1 (0.9%)	1.0

*ANA* antinuclear antibody; *AZA* azathioprine; *bDMARD* biological disease-modifying antirheumatic drug; *cDMARD* conventional disease-modifying antirheumatic drug; *CNS* central nervous system; *CRP* C-reactive protein; *ESR* erythrocyte sedimentation rate; *FMF* familial Mediterranean fever; *GIS* gastrointestinal system; *JIA* juvenile idiopathic arthritis (JIA); *PFAPA* periodic fever, aphthous, pharyngitis, and adenitis; *RD* rheumatic disease; *TINU* tubulointerstitial nephritis and uveitis

### Comparative analysis of the patients who needed biologic treatments and who did not

**Fig. 1:**
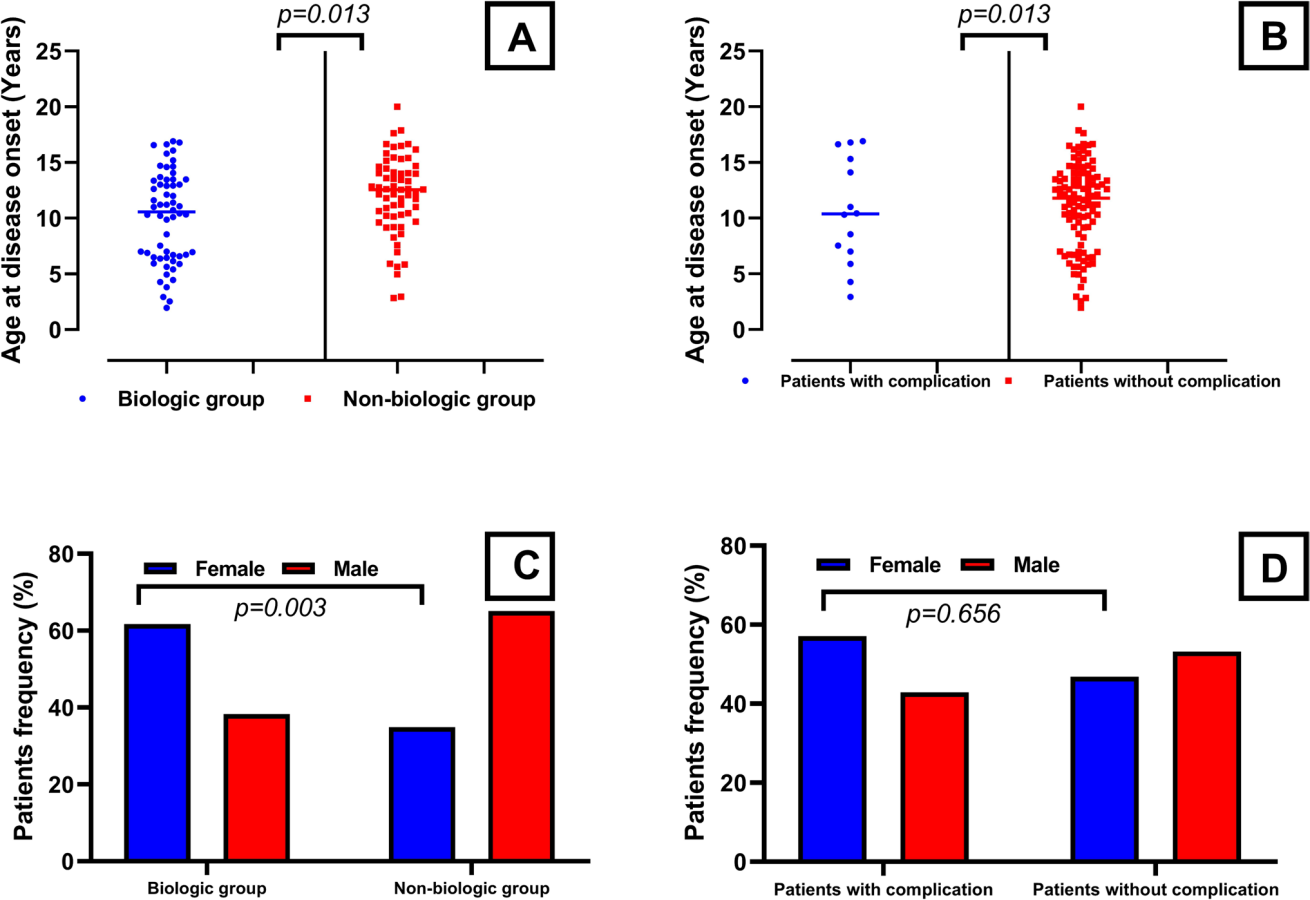
Comparison of age at disease onsets and gender frequencies of the patients according to biologic needing and ocular complication development. **A** Comparison of age at disease onsets between the patients required biological treatment and the patients did not. **B** Comparison of age at disease onsets between the patients experienced ocular complication and the patients did not. **C** Comparison of gender frequencies between the patients required biological treatment and the patients did not. **D** Comparison of gender frequencies between the patients experienced ocular complication and the patients did not

The need for biologic therapy was significantly higher in females as shown in Fig. 1 (61.7% vs. 34.9%; *p* = 0.003) and those requiring biologic therapy had a significantly younger age at onset of uveitis (10.57 (1.96–16.9) years *vs.* 12.56 (2.82–20.01) years; *p* = 0.013).Vascular and respiratory involvement signs were only observed in biologic group. Although the most common diagnosis among the biologic receivers was JIA, patients with BD had the highest percentage of patients who needed biologics. In the follow-up, ocular complications were significantly more common in patients who needed biologic treatments. Comparative analysis of both groups are presented in Table 3.

**Table 3:** Comparison data between patients with and without biological treatment needs

	Biological group (*n* = 60)	Non-biological group (*n* = 63)	*p* value
Female gender (*n*, %)	37 (61.7%)	22 (34.9%)	**0.003**
Age at diagnosis (years) (mean ± SD)	14.27 ± 4.85	15.49 ± 4.83	0.163
Uveitis			
Age at baseline (median (min–max))	10.57 (1.96–16.9)	12.56 (2.82–20.01)	**0.013**
Symptomatic (*n*, %)	47 (78.3%)	57 (90.5%)	0.107
Unilateral (*n*, %)	6 (10%)	16 (25.4%)	**0.046**
Bilateral (*n*, %)	54 (90%)	47 (74.6%)	**0.046**
Anterior (*n*, %)	42 (70%)	55 (87.3%)	**0.045**
Intermediate (*n*, %)	7 (11.7%)	4 (6.3%)	**0.045**
Posterior (*n*, %)	0 (0%)	1 (1.6%)	**0.045**
Panuveitis (*n*, %)	11 (18.3%)	3 (4.8%)	**0.045**
Extraocular finding (*n*, %)	26 (43.3%)	22 (34.9%)	0.441
Arthritis (*n*, %)	13 (21.7%)	8 (12.7%)	0.279
Mucocutaneous (*n*, %)	10 (16.7%)	11 (17.5%)	1.0
GIS (*n*, %)	1 (1.7%)	1 (1.6%)	0.972
CNS (*n*, %)	1 (1.7%)	1 (1.6%)	0.972
Renal (*n*, %)	4 (6.7%)	5 (7.9%)	1.0
Vascular (*n*, %)	2 (3.3%)	0 (0%)	0.144
Respiratory (*n*, %)	2 (3.3%)	0 (0%)	0.144
Rheumatic disease (*n*, %)	22 (36.7%)	13 (20.6%)	0.077
FMF (*n*, %)	1 (1.7%)	0 (0%)	0.304
PFAPA (*n*, %)	0 (0%)	1 (1.6%)	0.327
JIA (*n*, %)	12 (20%)	7 (11.1%)	0.265
Behçet (*n*, %)	6 (10%)	1 (1.6%)	0.058
TINU (*n*, %)	3 (5%)	5 (7.9%)	0.718
Sarcoidosis (*n*, %)	1 (1.7%)	0 (0%)	0.304
Family history			
Uveitis in the family (*n*, %)	3 (5%)	1 (1.6%)	0.357
RD in the family (*n*, %)	7 (11.7%)	4 (6.3%)	0.473
Laboratory			
ANA positivity (*n*, %)	18 (30%)	15 (23.8%)	0.568
ESR (median (min–max))	13.5 (2–91)	11 (2–71)	0.065
CRP (mg/L) (median (min–max))	2 (0.1–47)	3 (0.08–94)	0.514
Platelets (median (min–max))	297,500 (173,000–542,000)	284,000 (180,000–670,000)	0.161
Treatment			
Systemic steroids (*n*, %)	58 (96.7%)	26 (41.3%)	** < 0.01**
cDMARD (*n*, %)	60 (100%)	37 (58.7%)	** < 0.01**
Methotrexate (*n*, %)	55 (91.7%)	33 (52.4%)	** < 0.01**
Sulfasalazine (*n*, %)	3 (5%)	3 (4.8%)	1.0
AZA (*n*, %)	6 (10%)	4 (6.3%)	0.523
Cyclosporine (*n*, %)	2 (3.3%)	1 (1.6%)	0.613
Surgery (*n*, %)	6 (10%)	0 (0%)	**0.012**
Complication (*n*, %)	12 (20%)	2 (3.2%)	**0.008**
Band keratopathy (*n*, %)	3 (5%)	0 (0%)	0.113
Synechia (*n*, %)	1 (1.7%)	0 (0%)	0.304
Glaucoma (*n*, %)	2 (3.3%)	1 (1.6%)	0.613
Cataract (*n*, %)	9 (15%)	1 (1.6%)	**0.008**

*ANA* antinuclear antibody; *AZA* azathioprine; *cDMARD* conventional disease-modifying antirheumatic drug; *CNS* central nervous system; *CRP* C-reactive protein; *ESR* erythrocyte sedimentation rate; *FMF* familial Mediterranean fever; *GIS* gastrointestinal system; *JIA* juvenile idiopathic arthritis (JIA); *PFAPA* periodic fever, aphthous, pharyngitis, and adenitis; *RD* rheumatic disease; *TINU* tubulointerstitial nephritis and uveitis

### Risk factors of severe disease

Female gender (univariate: aOR 2.99, 95% C.I. 1.439–6.248, *p* = 0.003; multivariate: aOR 3.265, 95% C.I. 1.455–7.322, *p* = 0.004) and younger age at uveitis onset (univariate: aOR 0.875, 95% C.I. 0.795–0.965, *p* = 0.007; multivariate: aOR 0.846, 95% C.I. 0.759–0.944, *p* = 0.007) were strongly associated with increased need for biologics in both univariate and multivariate logistic regression analysis. Furthermore, BD was a strong risk factor for complications in both univariate (aOR, 14.133; 95% C.I. 2.765–72.231, *p* = 0.001) and multivariate (aOR 24.159, 95% C.I. 2.592–225,152, *p* = 0.005) logistic regression analysis (Table 4).

**Table 4:** Risk factors of severe disease

	Model 1: risk factors of biological treatment requirement*					
	Univariate analysis			Multivariate analysis		
	aOR	95% C.I	*p*	aOR	95% C.I	*p*
Female sex	2.998	1.439–6.248	0.003	3.265	1.455–7.322	0.004
Early-onset uveitis	0.875	0.795–0.965	0.007	0.846	0.759–0.944	0.003
Type of uveitis	1.468	0.859–2.509	0.160	1.780	0.984–3.222	0.057
RD	2.227	0.966–4.981	0.051	2.337	0.953–5.728	0.064
ANA positivity	1.371	0.616–3.054	0.439	1.017	0.413–2.508	0.970
	Model 2: risk factors of uveitis-related complications**					
	Univariate analysis			Multivariate analysis		
	aOR	95% C.I	*p*	aOR	95% C.I	*p*
Behçet disease	14.133	2.765–72.231	0.001	24.159	2.592–225.152	0.005
Early-onset uveitis	0.962	0.837–1.107	0.591	0.944	0.810–1.100	0.461
Female sex	1.516	0.493–4.663	0.468	2.290	0.631–8.319	0.208
RD	2.069	0.661–6.473	0.212	0.707	0.136–3.678	0.680
ANA positivity	0.718	0.187–2.754	0.629	0.748	0.178–3.152	0.693

*RD* rheumatologic disease

*Model fitting tests for model 1: Omnibus test—chi-square = 24.567, *p* = 0.000; Hosmer–Lemeshow test—chi-square = 4.657, *p* = 0.794; Nagelkerke *R* square = 0.241

**Model fitting tests for model 2: Omnibus test—chi-square = 11.841, *p* = 0.037; Hosmer–Lemeshow test—chi-square = 7.401, *p* = 0.494; Nagelkerke *R* square = 0.181

## Discussion

In this study, we analyzed the demographic and laboratory data, underlying systemic diseases, treatments administered, and uveitis-related complications experienced by our patients with non-infectious pediatric uveitis. Our study found that being female and early age at disease onset were significantly associated with an increased need for biologic treatments. Additionally, BD was strongly linked to uveitis-related complications. To build upon these findings, it is essential to delve deeper into the specific treatments and their outcomes, examining how these factors contribute to managing the disease and improving patient prognosis.

It is widely recognized that female gender is a risk element for JIA-related uveitis [17]. Nevertheless, the prevalence of childhood non-infectious uveitis among different genders remains ambiguous, as various studies have reported contradictory findings [18–21]. The male/female ratio in our cohort was 1.08:1 (64 male and 59 female). We attribute the balanced gender distribution in our cohort to the relatively low JIA rate. Previous pediatric uveitis studies of the gender distribution support to our study [20–22]. In a study conducted by Ozdal et al. [23], it was found that the higher prevalence of male gender was linked to the fact that BD was the most common comorbid systemic disease, which has been reported that BD is slightly more common in male children [24].

The most common type was anterior uveitis which was seen in almost four-fifth of the patients, consistent with the previous reports [22, 23]. Approximately one quarter of our patients had an underlying systemic rheumatologic disease. The most common rheumatologic disease was JIA, which was largely consistent with previous studies [19, 21, 25]. It is known that uveitis associated with JIA most commonly causes anterior uveitis [8, 26]. In approximately 80% of JIA patients in our cohort, the type of uveitis was anterior uveitis, which was consistent with the previous literature [26, 27]. However, it is important to note that the majority of patients with anterior uveitis do not have JIA. We suspect that some of these cases may be JIA cases that started with ocular involvement but did not have the chance to develop arthritis due to the frequent use of cDMARD and biologic drugs, which are also used in the treatment of arthritis in this patient group.

All our patients with BD had panuveitis. This data confirms the knowledge that Behçet’s disease-associated uveitis is typically characterized by panuveitis [17]. We also found that around 40% of our patients with BD did not report symptomatic disease. This might be related with the limited ability of the children to notice or report their ocular problems, unlike adults with the same disease. Therefore, we think that even if they have no ocular complaints, uveitis screening in patients with BD, as well as JIA, is crucial to capture these cases.

Although it is rare, uveitis was reported to be the second most common ocular involvement sign after the conjunctivitis in FMF patients [28]. One of our patients had with asymptomatic anterior uveitis had coexisting FMF and JIA. It remains uncertain whether the risk of uveitis changes in JIA with coexisting FMF. Besides, uveitis associated with PFAPA syndrome has been reported rarely. However, the exact frequency and mechanism are not clearly known [29]. One of our patients with isolated PFAPA had acute symptomatic anterior uveitis. Our findings are in line with the previously reported data that autoinflammatory diseases may be accompanied by red eye including conjunctivitis, episcleritis, and uveitis [30].

In our study, we found that patients with rheumatic disease had significantly higher ESR levels at presentation compared to patients with isolated uveitis. Although not statistically significant, patients with rheumatic disease also had higher CRP levels at presentation. In a study comparing patients with isolated uveitis, BD-related uveitis, and JIA-related uveitis, ESR values were found to be significantly higher in patients with rheumatologic diseases, similar to our data [20]. Consequently, we suggest that pediatric uveitis patients with high ESR values should undergo thorough examination for potential underlying rheumatologic diseases.

It is known that oligoarticular type and ANA positivity are risk factors for the development of uveitis in JIA patients [17]. Consistent with the literature data, we found that ANA was positive in almost half of the patients with JIA-related uveitis. In addition, approximately 2/3 of the ANA positive JIA patients had oligoarticular JIA.

Although all patients in our study received local glucocorticoids as first-line treatment, only in one fifth of our patient’s uveitis could be controlled with local treatment. The remaining patients needed cDMARDs. The most commonly prescribed cDMARD was methotrexate (MTX) and azathioprine (AZA) was the second one, similar to previous studies [20, 21]. Biologic therapy was needed in almost half of our patients. Anti-TNFs are the most commonly used biologic treatment agents in accordance with recommendations of treatment guidelines [9, 10].

To the best of our knowledge, this study demonstrated for the first time that the need for biologic treatments was significantly higher in female patients compared to male pediatric uveitis patients. Furthermore, the results of the binary logistic regression analysis indicated that being female was a risk factor for the necessity of biological treatment. It is known that females are at increased risk of autoimmune diseases and JIA-related uveitis [17, 26, 31]. Our analysis indicated that patients with rheumatologic diseases had a more pressing need for biological treatment compared to those with idiopathic uveitis. However, in the group with rheumatologic disease, there were 19 female and 16 male patients, with the numbers being relatively equal. Additionally, although we added the presence of rheumatic disease in to the regression analysis which was set for evaluate the risk factors for biologic requirement as a confounding factor, we found the female gender as an independent risk factor. Therefore, we suggest that female patients with uveitis should be closely monitored due to significantly increased biological treatment needing.

We showed that early disease onset is also a significant predictor of biological treatment requirement, as supported by previous [32]. Earlier disease onset may be due to a higher genetic burden that promotes autoimmune inflammation. These patients also have a longer exposure to chronic inflammation, which can transform into a form that is not easily controlled. Therefore, we think that biological treatment options may be considered in early stages of the diseases in patients with younger age of disease onset.

Pediatric uveitis is a condition that can result in a variety of complications, including cataract, synechiae, and glaucoma. The prevalence of these complications varies significantly between studies, ranging from 14.7% to 69% [18, 33–35]. In our study, 11.4% of the patients experienced complications, and the most common was cataract. The main factors that cause this variation in complication rated can be different distribution of systemic diseases in different cohorts, variations in disease duration, and easier access to biologic treatments.

The influence of gender was not thoroughly studied in pediatric uveitis patients. Here, we found a higher rate of uveitis-related complications in girls compared to boys; the complication rate in BD was also significantly higher and not affected by the confounding factors we identified. BD is typically characterized by bilateral, recurrent panuveitis [17]. Posterior segment involvement is associated with a higher risk of ocular complications, and all pediatric BD patients in our cohort had panuveitis similar to previous reports [12, 36]. Therefore, it is not surprising that this patient group had a higher complication rate. Our study additionally shows that BD is a significant risk factor for the development of ocular complications.

The main limitation of our study is that it was designed as a retrospective single-center study. Additionally, despite having a reasonable number of patients overall, we did not have a sufficient number of patients in the subgroups to conclude uveitis associated with rare rheumatologic diseases. Another noteworthy limitation of our study was that the follow-up period of at least 1 year may be inadequate to evaluate the progression of chronic conditions. On the other hand, the main strength of our study is that we evaluated a broad range of rheumatic diseases and analyzed the possible predictors of the need for biological treatments, complications, and risk factors for severe disease.

In conclusion, we found that an early age of uveitis onset and female gender are strongly associated with the need for biologic treatment, while BD is a strong predictor of uveitis-related complications. We think that female gender, those with early onset of disease, and patients with BD should be closely monitored due to the significantly increased risk of serious illness, and more caution should be exercised when prescribing treatment in these patients.

## Data Availability

All data relevant to the study are included in the article.

## Declarations

### The patient and public involvement statement

Patients or the public were not involved in the design, or conduct, or reporting, or dissemination plans of our research.
